# Incorporating Metastatic Disease Burden into Prognostic Assessment in Metastatic Castration-Resistant Prostate Cancer: The MIRA Score

**DOI:** 10.3390/cancers18162660

**Published:** 2026-08-18

**Authors:** Mario Uccello, Panagiotis J. Vlachostergios, Aruni Ghose, Stergios Boussios

**Affiliations:** 1 Royal United Hospitals Bath NHS Foundation Trust, Bath BA1 3NG, UK; mario_uccello@hotmail.it; 2 Department of Medical Oncology, IASO Thessalias Hospital, 41500 Larissa, Greece; 3 Department of Medical Oncology, Ioannina University Hospital, 45500 Ioannina, Greece; 4 Faculty of Medicine, School of Health Sciences, University of Ioannina, 45110 Ioannina, Greece; 5 Division of Hematology & Medical Oncology, Department of Medicine, Weill Cornell Medicine, New York, NY 10065, USA; 6 Cancer Division, University College London Hospitals NHS Foundation Trust, London NW1 2BU, UK; 7 Department of Research and Innovation, Medway NHS Foundation Trust, Gillingham ME7 5NY, UK; 8 School of Cancer and Pharmaceutical Sciences, Faculty of Life Sciences and Medicine, King’s College London, London SE1 1UL, UK; 9 Faculty of Medicine, Health and Social Care, Canterbury Christ Church University, Canterbury CT1 1QU, UK; 10 AELIA Organisation, 9Th Km Thessaloniki-Thermi, 57001 Thessaloniki, Greece

**Keywords:** metastatic castration-resistant prostate cancer, tumour burden, prognostic assessment, risk stratification, overall survival

## Abstract

**Background:** Prognostic assessment in metastatic castration-resistant prostate cancer (mCRPC) remains important, but metastatic burden is not routinely captured as a simple combined measure. This exploratory study developed the Metastatic Integrated Risk Assessment (MIRA) score using disease burden and routine variables. **Methods:** Anonymised placebo-arm individual patient data from D4320C00014/ENTHUSE-M1 were analysed. Baseline variables were assessed for association with overall survival (OS) using Cox regression. A hierarchical composite tumour-burden variable combined bone-metastasis categories with RECIST target-lesion presence and sum of longest diameters (SLD). Selected variables formed an additive score, stratified into three risk groups, and were compared with the reconstructed Halabi classification using Harrell’s C-index. The final multivariate Cox model underwent internal validation using 1000 bootstrap resamples. **Results:** Among 266 placebo-treated patients, 133 deaths occurred, and median OS was 22.21 months. Composite tumour burden showed the strongest prognostic association and remained independently associated with OS. MIRA risk groups showed clear OS separation. In this derivation cohort, MIRA showed higher apparent discrimination than the reconstructed Halabi classification, with C-indices of 0.755 and 0.646, respectively; the paired difference was statistically significant (*p* < 0.001). For the final multivariate Cox model, the apparent C-index was 0.779, and the optimism-corrected C-index was 0.760; the optimism-corrected calibration slope was 0.841. **Conclusions:** These exploratory findings support metastatic tumour burden as a relevant prognostic factor in mCRPC and suggest that a simple combined measure of skeletal and measurable soft-tissue disease may have value for risk stratification. Refinement and independent external validation of MIRA are required before clinical use.

## Simple Summary

People with advanced prostate cancer can have very different survival outcomes, even when their disease appears similar. This exploratory study developed a tool, called MIRA, to estimate survival in men whose prostate cancer had spread and had become resistant to standard hormone treatment. Researchers analysed anonymised data from 266 men in the placebo group of a historical clinical trial. The score combined the amount of cancer in the bones and other measurable sites, with information including age, pain, speed of disease progression, inflammation and blood test results. Patients were divided into three different prognostic groups. In this dataset, the MIRA groups performed better than an established tool, called the Halabi classification. The findings suggest that measuring the overall amount of cancer may improve prognostic assessment. However, MIRA must be refined and tested in contemporary patient populations before its use in clinical practice.

## 1. Introduction

Survival in metastatic castration-resistant prostate cancer (mCRPC) is highly variable. Patients with the same broad disease state may differ substantially in metastatic extent, symptom burden, prior treatment history, systemic inflammation, laboratory profile and physiological reserve. Prognostic assessment remains important for counselling, trial stratification and interpretation of survival outcomes [1,2]. Several prognostic models have been developed in advanced prostate cancer, including the Smaletz–Kattan nomogram [3], the TAX327 nomogram [4], Halabi prognostic models [1,5,6], Templeton et al.’s [7] simple prognostic score, incorporating the neutrophil-to-lymphocyte ratio (NLR), Chi et al.’s [8] prognostic index model for abiraterone-treated mCRPC, the PREVAIL Model for Prostate Cancer Survival [2] and the Prostate Cancer DREAM Challenge model [9]. Across these approaches, recurrent adverse prognostic domains include performance status, pain, anaemia, alkaline phosphatase (ALP), lactate dehydrogenase (LDH), prostate-specific antigen (PSA), metastatic site, inflammatory status and opioid use. However, some models require weighted calculations, are anchored to specific treatment settings, or depend on more complex analytic methods. This may limit their use as simple, transparent risk classifications across heterogeneous clinical-trial and real-world datasets [1,2,8]. Metastatic disease burden is one of the most clinically apparent features of advanced cancer, but it is not always captured in a practical or consistent form in mCRPC prognostic models [1,8,10]. This is particularly relevant in prostate cancer, where metastatic involvement is commonly skeletal and may not be fully represented by conventional measurable-disease criteria. A prognostic score that incorporates both skeletal and measurable soft-tissue disease burden, together with routinely available clinical and laboratory variables, may provide a reproducible approach to risk stratification without requiring specialised modelling infrastructure or molecular data. The Metastatic Integrated Risk Assessment for the mCRPC (MIRA) score was developed using anonymised placebo-arm patient-level data from the ENTHUSE-M1 trial [11]. The aim was to construct a simple prognostic score based on routinely available baseline variables, with explicit incorporation of metastatic disease burden, and to compare its discriminatory performance with the established Halabi classification.

## 2. Materials and Methods

### 2.1. Data Source and Study Population

This was a secondary analysis of anonymised individual patient-level data accessed through Project Data Sphere [12]. The analysis used the available placebo-arm dataset from D4320C00014/ENTHUSE-M1 [11], a phase III, randomised, double-blind, placebo-controlled trial evaluating ZD4054 versus placebo in men with hormone-resistant prostate cancer and bone metastases who were pain-free or mildly symptomatic. Only patients allocated to placebo were included, as these were the only patient-level data available for the present analysis. Available study documentation, including the clinical study protocol, case report form, data files and accompanying format files, was reviewed to identify relevant baseline clinical, laboratory, patient-reported and imaging variables. Overall survival (OS) was defined as the time from randomisation to death from any cause. Patients alive at last follow-up were censored.

### 2.2. Variables and Data Preparation

Baseline demographic, clinical, laboratory, patient-reported and imaging variables were extracted and harmonised at patient level. Laboratory variables were based on baseline values. In rare cases where a baseline laboratory value was missing, the next available value was used; this was considered acceptable in the context of a placebo-arm prognostic analysis. The frequency and timing of these substitutions were quantified for each laboratory variable. Candidate predictors were identified from the available baseline variables on the basis of established or plausible prognostic relevance in mCRPC, routine clinical availability and reproducibility. Clinically established or dataset-prespecified cut-offs were used rather than thresholds optimised against OS in the present cohort. For the present exploratory analysis, a hierarchical composite tumour-burden variable was developed using both the number of bone metastases and Response Evaluation Criteria in Solid Tumours (RECIST) target-lesion burden. The bone-metastasis categories already recorded in the dataset, including <5, 5–20 and ≥21 lesions, were used, while the RECIST sum of the longest diameters (SLD) threshold of >3 cm was selected because 3 cm corresponded to the first tertile of the observed SLD distribution among patients with RECIST target lesions. Composite tumour-burden level was assigned hierarchically. Level 2 was assigned to patients with ≥21 bone metastases or RECIST SLD > 3 cm. Among the remaining patients, level 1 was assigned to those with 5–20 bone metastases or any RECIST target lesion. Level 0 was assigned to those with <5 bone metastases and no RECIST target lesion. Other evaluated variables included age, race, baseline World Health Organization (WHO)/Eastern Cooperative Oncology Group (ECOG) performance status, time from first oncological therapy, NLR, ALP, haemoglobin (Hb), PSA, LDH, albumin and platelet count. Baseline symptom burden was assessed using the Brief Pain Inventory (BPI) and the EQ-5D pain/discomfort dimension; the selected thresholds were BPI ≥ 1 and EQ-5D pain/discomfort ≥ 2. The composite was considered positive if either threshold was met. For patients with one of the two measures unavailable, the other was used; no patient had both measures missing, so the composite BPI/EQ-5D variable was available for all 266 patients. When one or more MIRA components were missing, the exact total score was considered unavailable. Risk-group assignment was retained when the available components were sufficient to determine the category unequivocally.

### 2.3. Statistical Analysis

Associations between candidate variables and OS were first assessed using univariate Cox proportional-hazards analysis. Variables considered for multivariate modelling and score construction were selected manually, rather than through a formal automated procedure, according to statistical evidence, clinical interpretability, established prognostic relevance and ease of reproducibility. Variables independently associated with OS were prioritised, while variables with borderline adjusted associations or strong univariate associations were also considered when routinely available and clinically meaningful. Particular attention was given to tumour burden because of its expected prognostic relevance. Opioid use at baseline was available and was used for reconstruction of the Halabi score, but it was not evaluated as a candidate prognostic variable for the MIRA score because it was reported in only five patients. The MIRA score for mCRPC was constructed as an additive point-based prognostic score. The resulting score included age, symptom burden assessed by BPI/EQ-5D, composite tumour-burden level, NLR and time since first prior oncological therapy, together with ALP, Hb and PSA, which were retained because of their established prognostic relevance and strong univariate or borderline multivariate associations in this cohort. Among laboratory variables not independently associated with OS, PSA was retained because of its stronger univariate association, disease-specific prognostic relevance and greater likelihood of routine availability across future clinical and research datasets, whereas LDH showed a weaker univariate association in this cohort and was excluded to preserve parsimony and reproducibility. Point allocation was pragmatic rather than derived by formal scaling or rounding of the Cox regression coefficients. The hierarchical tumour-burden component was weighted progressively according to increasing disease burden, while time since first prior oncological therapy <2 years was assigned two points to reflect its adverse prognostic association and its interpretation as a marker of aggressive disease tempo. Age ≥ 80 years was deliberately limited to one point despite the strength of its association with OS, because chronological age was considered a non-specific host factor and a potential surrogate for more direct measures of comorbidity, frailty, physiological reserve and functional status. All other predictors were assigned one point to preserve the simplicity of the score. OS according to MIRA risk group was estimated using the Kaplan–Meier method and compared using the log-rank test. Cox proportional-hazards models were used to estimate hazard ratios (HRs) and 95% confidence intervals (CIs), with the low-risk group used as the reference category. For benchmarking, the updated Halabi prognostic score for metastatic castration-resistant prostate cancer was reconstructed using available baseline variables from the placebo dataset, including opioid analgesic use, in accordance with the published 2014 model [1]. Patients were classified according to the published three-tier Halabi risk-group classification, allowing direct comparison with MIRA. Discrimination was assessed using Harrell’s C-index. C-indices for the two classifications were compared using the non-parametric method for two correlated C-indices with right-censored survival outcomes proposed by Kang et al. [13]. This method accounts for the paired nature of the comparison, because both classifications were evaluated in the same patients, and estimates the variance of the difference between C-indices using an asymptotic normal approximation. As a sensitivity analysis, the MIRA risk-group Cox model and Harrell’s C-index were recalculated after excluding patients for whom a post-baseline laboratory measurement had been used for at least one score component. The final multivariate Cox model included 10 predictors. Because the three-level composite tumour-burden variable required two coefficients, 11 regression parameters were estimated in total. With 131 deaths, this corresponded to approximately 11.9 events per parameter. The number of regression parameters was limited relative to the number of observed events to reduce the risk of overfitting. The proportional-hazards assumption was assessed for the final multivariate Cox model and the MIRA risk-group Cox model using scaled Schoenfeld residuals with the Kaplan–Meier transformation of time. Where covariate-specific evidence of non-proportionality was observed, a sensitivity analysis incorporating time-dependent effects was performed. Internal validation of the final multivariate Cox model was performed using 1000 bootstrap resamples. The complete-case analysis dataset used to fit the final multivariate model was resampled with replacement, and the same final set of covariates was refitted in each bootstrap sample. Optimism was estimated by comparing model discrimination within each bootstrap sample with its performance when applied to the original dataset. Mean optimism was then subtracted from the apparent C-index to obtain the optimism-corrected C-index. An optimism-corrected calibration slope and bootstrap 95% CIs were also estimated. Calibration of predicted survival probabilities was assessed at 12 and 24 months using bootstrap-corrected calibration plots comparing predicted with observed survival. Risk groups were coded so that higher values indicated higher mortality risk. Two-sided *p* values were reported, with *p* < 0.05 considered statistically significant. C-indices and their confidence intervals were reported to three decimal places. All statistical analyses were performed using MedCalc Statistical Software version 23.6.1, R version 4.6.1 and RStudio version 2026.04.0 + 526, with the survival, compareC and rms packages used where appropriate.

## 3. Results

### 3.1. Patient Characteristics and Baseline Tumour Burden

The cohort included 266 placebo-assigned patients with hormone-resistant prostate cancer and bone metastases. At data cut-off, 133 deaths had occurred and 133 patients were censored. Median OS was 22.21 months (95% CI, 18.76–23.23). Baseline demographic, clinical, laboratory and disease-burden characteristics are summarised in Table 1. Missing baseline laboratory values were infrequent. The earliest post-baseline measurement was used for one to seven patients per variable (0.4–2.6%), while one platelet value remained unavailable. The frequency and timing of these substitutions are reported in Supplementary Table S1. Overall, 43 patients (16.2%) were aged ≥80 years and 70 (26.3%) had a WHO/ECOG performance status 1. Although patients were pain-free or mildly symptomatic at trial entry, baseline patient-reported measures identified a measurable symptom burden: BPI worst pain ≥1 was recorded in 132 of 258 evaluable patients (51.2%), and EQ-5D pain/discomfort level 2 in 103 of 258 (39.9%). The composite BPI/EQ-5D variable was positive in 153 of 266 patients (57.5%). Overall, 189 patients (71.1%) had ≥5 bone metastases and 53 (19.9%) had ≥21 bone metastases. A total of 265 patients were evaluable for visceral disease, nodal disease, RECIST target lesions and composite tumour-burden level. Visceral metastases were documented in 45 patients (17.0%) and nodal metastases in 80 (30.2%). RECIST target lesions were present in 94 patients (35.5%), with a RECIST SLD > 3 cm in 58 (21.9%). Composite tumour-burden level was classified as level 0 in 52 patients (19.6%), level 1 in 120 (45.3%) and level 2 in 93 (35.1%).

### 3.2. Univariate and Multivariate Associations with OS

In univariate Cox regression analysis, several demographic, clinical, laboratory and disease-burden variables were associated with OS (Table 2). These included age ≥80 years, a positive BPI/EQ-5D composite, time since first prior oncological therapy <2 years, Hb < 120 g/L, NLR > 2.5, albumin ≤ 40 g/L, ALP > 130 U/L, LDH > 240 U/L, PSA ≥ 100 ng/mL, higher bone-metastatic burden, nodal metastases, presence of RECIST target lesions, RECIST SLD > 3 cm and higher composite tumour-burden level. The strongest univariate associations were observed for composite tumour-burden level, with HRs of 4.35 (95% CI, 2.04–9.27) for level 1 and 10.38 (95% CI, 4.88–22.09) for level 2, compared with level 0. A multivariate Cox model including composite tumour-burden level and candidate clinical and laboratory variables was fitted in 264 patients with complete covariate data, including 131 deaths (Table 3). The overall model was statistically significant (χ^2^ = 115.806, df = 11, *p* < 0.001), with a Harrell’s C-index of 0.779 (95% CI, 0.741–0.818). In this model, composite tumour-burden level remained independently associated with OS: compared with level 0, HRs were 2.62 (95% CI, 1.20–5.69; *p* = 0.015) for level 1 and 5.52 (95% CI, 2.52–12.11; *p* < 0.001) for level 2. Other variables independently associated with OS were age ≥80 years, a positive BPI/EQ-5D composite, time since first prior oncological therapy <2 years and NLR > 2.5. Hb < 120 g/L and ALP > 130 U/L showed borderline associations, whereas albumin ≤ 40 g/L, LDH > 240 U/L and PSA ≥ 100 ng/mL were not independently associated with overall survival after adjustment. The proportional-hazards assumption was not violated globally for the final multivariate Cox model (global Schoenfeld test, χ^2^ = 10.90, df = 11, *p* = 0.453). Covariate-specific tests indicated possible non-proportionality for Hb < 120 g/L (*p* = 0.041) and PSA ≥ 100 ng/mL (*p* = 0.021). In a sensitivity analysis incorporating time-dependent effects for Hb and PSA, model fit was not significantly improved (likelihood-ratio χ^2^ = 5.84, df = 2, *p* = 0.054), and discrimination was essentially unchanged (Harrell’s C-index, 0.779 versus 0.779). Internal validation of the final multivariate Cox model using 1000 bootstrap resamples estimated a mean optimism of 0.020 in Harrell’s C-index. The apparent C-index of 0.779 was reduced to an optimism-corrected C-index of 0.760 (bootstrap 95% CI, 0.720–0.800). The optimism-corrected calibration slope was 0.841 (95% CI, 0.555–1.138), indicating modest evidence of overfitting. Bootstrap-corrected calibration plots at 12 and 24 months are shown in Figure S1. At 12 months, predicted and observed survival probabilities were closely aligned over most of the range, with some deviations at intermediate predicted probabilities; 194 patients remained at risk. At 24 months, greater deviations between predicted and observed survival were observed, and 37 patients remained at risk.

### 3.3. Development and Performance of the MIRA Score

**Figure 1:**
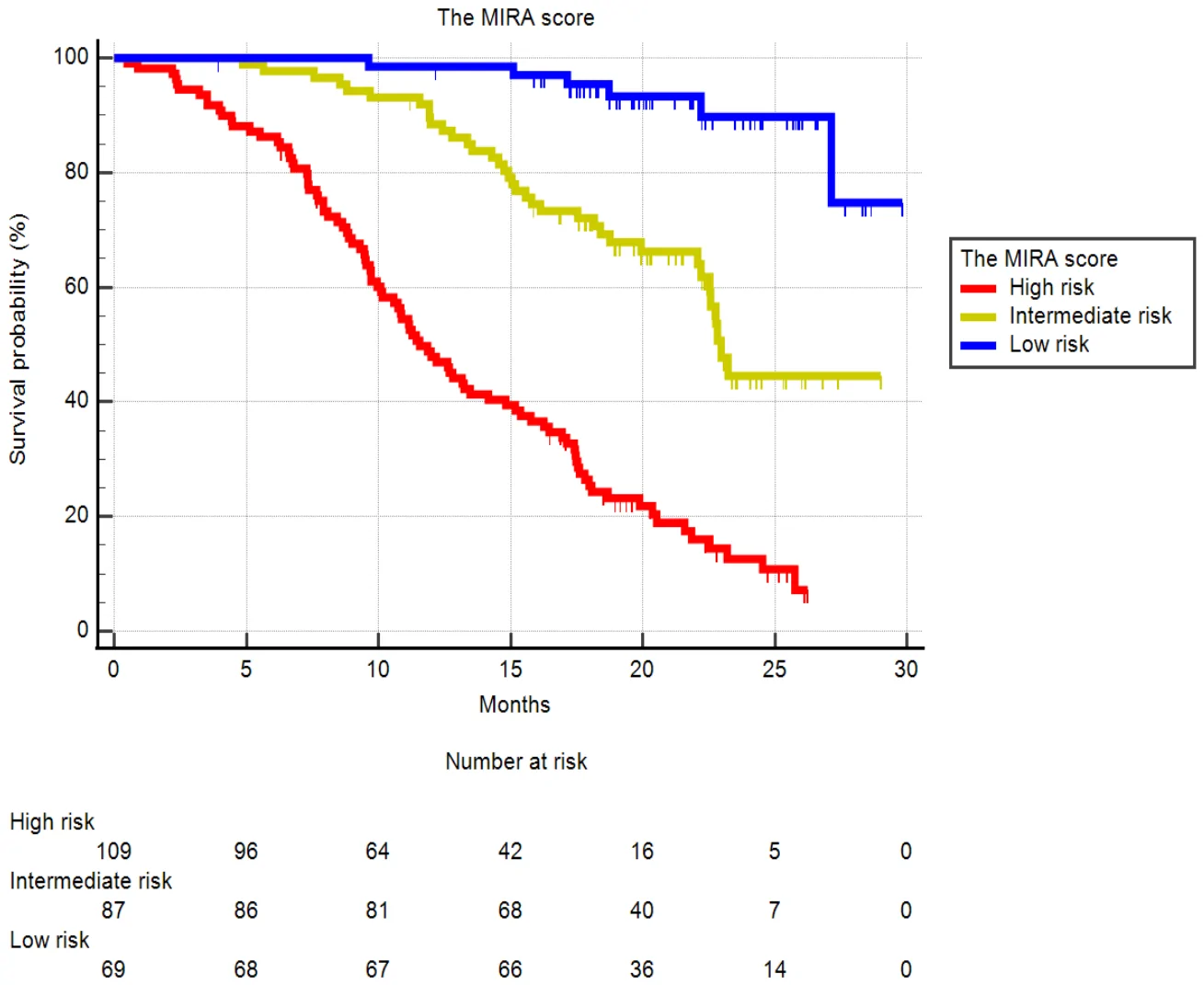
Overall survival (OS) according to the Metastatic Integrated Risk Assessment (MIRA) risk group. The Kaplan–Meier curves show OS according to MIRA risk classification. Median OS was not reached in the low-risk group, 22.97 months in the intermediate-risk group and 11.56 months in the high-risk group. Tick marks indicate censored observations, and the number of patients at risk is shown below the plot. OS distributions differed significantly across the three groups by the log-rank test (*p* < 0.001).

The MIRA score included eight variables selected as described in the Methods. The components and point allocation are presented in Table 4. The resulting score had a theoretical range of 0–10 points and an observed range of 0–9 points. In the 265 patients evaluable for risk-group classification, Kaplan–Meier analysis showed clear separation of OS across the low-, intermediate- and high-risk MIRA groups (Figure 1; log-rank *p* < 0.001). Median OS was not reached in the low-risk group, was 22.97 months (95% CI, 22.21–23.23) in the intermediate-risk group and 11.56 months (95% CI, 10.09–14.16) in the high-risk group (Table 5). The proportional-hazards assumption was not significantly violated for the MIRA risk-group Cox model (global Schoenfeld test, χ^2^ = 5.27, df = 2, *p* = 0.072). The MIRA score was compared with the Halabi score using the three-tier risk-group classification as the comparator. Survival also differed significantly across Halabi risk groups (log-rank *p* < 0.001), but discrimination was lower than with MIRA. Harrell’s C-index was 0.755 (95% CI, 0.722–0.788) for the MIRA classification and 0.646 (95% CI, 0.603–0.690) for the Halabi classification (Table 5). The absolute C-index improvement with MIRA was 0.110, and paired comparison confirmed that this difference was statistically significant (z = 4.30, *p* < 0.001). In a sensitivity analysis excluding the six patients with post-baseline substitution for at least one MIRA component, results were materially unchanged. Harrell’s C-index was 0.752 (95% CI, 0.719–0.785), compared with 0.755 in the primary analysis, and both the intermediate- and high-risk groups remained significantly associated with shorter OS (HR, 5.51 and 20.36, respectively; both *p* < 0.001).

## 4. Discussion

In this exploratory secondary analysis of the placebo arm of the ENTHUSE-M1 trial [11], we developed the MIRA score for mCRPC, a simple three-tier prognostic classification that showed clear separation of OS. Patients classified as low, intermediate and high risk showed markedly different survival trajectories, with a strong gradient in mortality across groups. The score was based entirely on baseline variables that are routinely available in clinical-trial or real-world datasets: tumour burden, age, symptom burden, prior treatment interval, inflammatory status and standard laboratory parameters. Importantly, the strongest signal was observed for baseline tumour burden, supporting the concept that the extent of metastatic disease remains a major determinant of the natural history of mCRPC, even in a population that was pain-free or only mildly symptomatic at study entry. The comparison with the Halabi classification [1] provides a clinically relevant benchmark. In this dataset, the Halabi three-tier classification remained prognostic, confirming its value as an established reference model in mCRPC. Although its C-index was lower than that of MIRA (0.646 versus 0.755), the Halabi classification was retrospectively reconstructed using the variables available in ENTHUSE-M1, with opioid use recorded in only five patients. In addition, MIRA was developed and evaluated in the same cohort. The observed difference in C-index should therefore be regarded as exploratory and not as evidence of superiority. In contrast to weighted nomograms or algorithmically derived models, the MIRA score was constructed as a transparent point-based score. Its purpose is not to replace more detailed prognostic tools in all settings, but to test whether a simple score incorporating tumour burden can retain prognostic relevance. As a prognostic rather than predictive model, MIRA should not currently be used to select treatment or determine surveillance intensity; following independent validation, it could potentially support prognostic counselling and clinical-trial stratification. The strong prognostic signal associated with tumour burden observed in this study is central to the interpretation of the MIRA score. The composite tumour-burden variable integrated bone-metastasis category and RECIST target-lesion burden, thereby capturing both skeletal disease extent and measurable soft-tissue disease. It was the dominant predictor in univariate analysis and remained independently associated with OS after adjustment for clinical and laboratory factors. Tumour burden is a recognised prognostic determinant across solid tumours [10,14], reflecting both extent of disease and underlying tumour biology. In prostate cancer, this concept has been formalised in the hormone-sensitive setting by classifications such as CHAARTED volume [15] and LATITUDE risk [16], which use metastatic extent and distribution to define clinically distinct risk groups. The present findings suggest that the same principle remains relevant after transition to castration resistance, even in patients who are pain-free or only mildly symptomatic. The interval from first oncological therapy to castration resistance also contributed substantially to risk stratification. A short interval to castration resistance is an established adverse prognostic marker and reflects rapid disease progression and more aggressive tumour biology [8,17]. It provides information complementary to baseline tumour burden, capturing disease tempo rather than disease extent at enrolment. In MIRA, the allocation of two points to this variable reflected both its independent association with OS and its clinical interpretation as a marker of aggressive disease tempo, rather than formal scaling of the regression coefficient. The historical context of ENTHUSE-M1 is an important limitation but also helps define how the findings should be interpreted. The trial was conducted before the current therapeutic era in mCRPC; therefore, the cohort cannot be considered fully representative of contemporary practice. However, patients were pain-free or only mildly symptomatic at study entry, making the population closer to chemotherapy-naïve mCRPC cohorts such as COU-AA-302 [18] and PREVAIL [19] than to later-line, highly symptomatic populations. This restricted clinical spectrum may explain why WHO/ECOG performance status, opioid analgesic use and the derived Halabi score had limited discriminatory value in the present dataset. Their prognostic contribution is expected to be greater in cohorts including patients with more advanced disease. The inflammatory component of MIRA was based on NLR, which has the advantage of being simple, inexpensive and widely available, while reflecting the balance between systemic inflammation and host immune status [7,20]. Alternative indices such as platelet-to-lymphocyte ratio or systemic immune-inflammation index may also be prognostic, but their reliance on platelet count can complicate interpretation [21,22]. In prostate cancer, both thrombocytopenia and thrombocytosis may reflect adverse biology or clinical vulnerability, including marrow involvement, systemic inflammation, frailty or treatment-related risk [23,24]. For a simple point-based score, NLR may therefore be preferable to platelet-based composite indices. The association between age and survival also deserves consideration. Age ≥ 80 years may reflect not only reduced background life expectancy, but also lower physiological reserve, competing comorbidity, vulnerability to intercurrent illness and reduced eligibility for subsequent systemic therapy, including chemotherapy. This distinction is important because chronological age is mainly an indirect surrogate for fitness [25]. Future versions of MIRA could assess whether age should be complemented or replaced by validated measures of comorbidity and frailty, such as the Charlson Comorbidity Index or the G8 geriatric screening tool, which may capture clinical vulnerability more accurately than age alone [26,27]. Several limitations should be acknowledged. First, this was a secondary analysis of a historical clinical-trial dataset and the score was developed and evaluated within the same cohort. Although bootstrap internal validation of the final multivariate Cox model suggested only modest optimism, the procedure did not reproduce the complete process of variable selection, point allocation and risk-group definition. The apparent performance of the MIRA classification may therefore retain residual optimism. Calibration was generally acceptable at 12 months. The greater deviations observed at 24 months should be interpreted cautiously because only 37 patients remained at risk, resulting in reduced precision of the longer-term calibration assessment. MIRA should consequently be regarded as a hypothesis-generating prognostic score within this cohort, and independent external validation and potential recalibration are essential before clinical use. Second, only placebo-arm patient-level data were available, limiting sample size and precluding assessment of whether the score behaves similarly in patients receiving active treatment. Third, ENTHUSE-M1 enrolled a selected, relatively favourable population with bone-metastatic, pain-free or mildly symptomatic mCRPC and WHO/ECOG performance status 0–1; visceral metastases were present in only 17% of evaluable patients. Consequently, the generalisability of the findings to broader contemporary mCRPC populations is uncertain, particularly for patients with poorer performance status, clinically significant pain, opioid requirements, frailty or more extensive visceral disease. Fourth, the dataset predates contemporary imaging and treatment pathways; androgen receptor pathway inhibitor sequencing, poly(ADP-ribose) polymerase (PARP) inhibitor eligibility, radioligand therapy, PSMA PET staging, genomic testing and circulating tumour DNA assessment were not represented. These advances may improve survival outcomes and the prognostic relevance of individual variables; therefore, MIRA should not be assumed to apply directly to patients treated in current practice. Following independent validation, MIRA could potentially support prognostic counselling and clinical-trial stratification in mCRPC.

## 5. Conclusions

In this exploratory secondary analysis, metastatic tumour burden emerged as a major prognostic determinant in mCRPC, and its integration with routinely available clinical and laboratory variables provided a pragmatic three-tier MIRA classification with clear separation of OS. MIRA should be regarded as a hypothesis-generating prognostic score because it was developed and evaluated within the same historical, selected placebo-arm cohort. Its performance therefore needs to be confirmed in contemporary mCRPC populations before clinical use. Future studies should also assess whether the prognostic performance of MIRA can be improved by integrating quantitative disease-burden measures with genomic and molecular biomarkers, advanced imaging and machine-learning approaches, while preserving transparency, clinical usability and responsible implementation [28,29,30].

## Figures and Tables

**Table 1:** Baseline characteristics and candidate prognostic variables.

Variable	Category	n/N (%)	Variable	Category	n/N (%)
**Demographic and clinical characteristics**			**NLR**	≤2.5	110/266 (41.4)
**Race**	White	160/266 (60.2)		>2.5	156/266 (58.6)
	Asian	99/266 (37.2)	**Albumin**	>40 g/L	202/266 (75.9)
	Other/unknown	7/266 (2.6)		≤40 g/L	64/266 (24.1)
**Age**	45–59 years	29/266 (10.9)	**ALP**	≤130 U/L	135/266 (50.8)
	60–64 years	29/266 (10.9)		>130 U/L	131/266 (49.2)
	65–69 years	54/266 (20.3)	**LDH**	≤240 U/L	226/266 (85.0)
	70–74 years	57/266 (21.4)		>240 U/L	40/266 (15.0)
	75–79 years	54/266 (20.3)	**PSA**	<100 ng/mL	176/266 (66.2)
	≥80 years	43/266 (16.2)		≥100 ng/mL	90/266 (33.8)
**BMI**	≥25 kg/m^2^	162/266 (60.9)	**Disease-burden variables**		
	<25 kg/m^2^	104/266 (39.1)	**Bone metastases**	<5	77/266 (28.9)
**WHO/ECOG PS**	0	196/266 (73.7)		5–20	136/266 (51.1)
	1	70/266 (26.3)		≥21	53/266 (19.9)
**Opioid use at baseline**	No	261/266 (98.1)	**Visceral metastases**	Absent	220/265 (83.0)
	Yes	5/266 (1.9)		Present	45/265 (17.0)
**BPI worst pain**	0	126/258 (48.8)	**Nodal metastases**	Absent	185/265 (69.8)
	≥1	132/258 (51.2)		Present	80/265 (30.2)
**EQ-5D pain/discomfort**	Level 1	155/258 (60.1)	**RECIST target lesion**	Absent	171/265 (64.5)
	Level 2	103/258 (39.9)		Present	94/265 (35.5)
**BPI/EQ-5D composite**	Negative	113/266 (42.5)	**RECIST SLD**	≤3 cm	207/265 (78.1)
	Positive	153/266 (57.5)		>3 cm	58/265 (21.9)
**Time since first prior oncological therapy**	≥2 years	191/265 (72.1)	**Composite tumour-burden level**	Level 0	52/265 (19.6)
	<2 years	74/265 (27.9)		Level 1	120/265 (45.3)
**Laboratory variables**				Level 2	93/265 (35.1)
**Hb**	≥120 g/L	208/266 (78.2)			
	<120 g/L	58/266 (21.8)			
**PLT**	150–450 × 10^9^/L	236/264 (89.4)			
	<150 or >450 × 10^9^/L	28/264 (10.6)			

Note: Percentages were calculated using evaluable patients for each variable. **Abbreviations:** ALP, alkaline phosphatase; BMI, body mass index; BPI, Brief Pain Inventory; ECOG, Eastern Cooperative Oncology Group; EQ-5D, EuroQol 5-Dimension questionnaire; Hb, haemoglobin; LDH, lactate dehydrogenase; NLR, neutrophil-to-lymphocyte ratio; PLT, platelet count; PSA, prostate-specific antigen; RECIST, Response Evaluation Criteria in Solid Tumours; SLD, sum of longest diameters; WHO, World Health Organization.

**Table 2:** Univariate Cox regression analysis of candidate prognostic variables for survival.

Variable	Category	Events/Total	HR (95% CI)	*p* Value
**Demographic and clinical variables**				
**Race**	Asian	51/99	1.17 (0.82–1.67)	0.38
	White	78/160		
**Age**	≥80 years	30/43	2.11 (1.40–3.18)	<0.001
	<80 years	103/223		
**BMI**	<25 kg/m^2^	59/104	1.40 (0.99–1.97)	0.06
	≥25 kg/m^2^	74/162		
**WHO/ECOG PS**	1	38/70	1.34 (0.92–1.95)	0.13
	0	95/196		
**BPI/EQ-5D composite**	Positive	87/153	1.74 (1.21–2.48)	0.003
	Negative	46/113		
**Time since first prior oncological therapy**	<2 years	53/74	2.45 (1.73–3.47)	<0.001
	≥2 years	79/191		
**Laboratory variables**				
**Hb**	<120 g/L	42/58	2.42 (1.67–3.49)	<0.001
	≥120 g/L	91/208		
**PLT**	<150 or >450 × 10^9^/L	15/28	1.50 (0.87–2.58)	0.14
	150–450 × 10^9^/L	116/236		
**NLR**	>2.5	91/156	1.79 (1.24–2.58)	0.002
	≤2.5	42/110		
**Albumin**	≤40 g/L	41/64	1.71 (1.18–2.47)	0.005
	>40 g/L	92/202		
**ALP**	>130 U/L	84/131	2.03 (1.42–2.89)	<0.001
	≤130 U/L	49/135		
**LDH**	>240 U/L	26/40	1.72 (1.12–2.64)	0.014
	≤240 U/L	107/226		
**PSA**	≥100 ng/mL	63/90	2.53 (1.80–3.56)	<0.001
	<100 ng/mL	70/176		
**Disease-burden variables**				
**Bone metastases**	≥21	42/53	5.03 (2.93–8.63)	<0.001
	5–20	71/136	2.52 (1.52–4.16)	<0.001
	<5	20/77		
**Visceral metastases**	Present	27/45	1.49 (0.97–2.27)	0.07
	Absent	105/220		
**Nodal metastases**	Present	50/80	1.78 (1.25–2.53)	0.001
	Absent	82/185		
**RECIST target lesion**	Present	60/94	2.24 (1.58–3.17)	<0.001
	Absent	72/171		
**RECIST SLD**	>3 cm	42/58	2.69 (1.86–3.90)	<0.001
	≤3 cm	90/207		
**Composite tumour-burden level**	Level 2	69/93	10.38 (4.88–22.09)	<0.001
	Level 1	55/120	4.35 (2.04–9.27)	<0.001
	Level 0	8/52		

HRs were estimated using univariate Cox proportional-hazards models. For bone-metastasis category and composite tumour-burden level, the reference categories were <5 bone metastases and level 0, respectively. Race was analysed as Asian versus White; patients recorded as other/unknown race were excluded from this comparison. **Abbreviations:** ALP, alkaline phosphatase; BMI, body mass index; BPI, Brief Pain Inventory; CI, confidence interval; ECOG, Eastern Cooperative Oncology Group; EQ-5D, EuroQol 5-Dimension questionnaire; Hb, haemoglobin; HR, hazard ratio; LDH, lactate dehydrogenase; NLR, neutrophil-to-lymphocyte ratio; PLT, platelet count; PSA, prostate-specific antigen; PS, performance status; RECIST, Response Evaluation Criteria in Solid Tumours; SLD, sum of longest diameters; WHO, World Health Organization.

**Table 3:** Multivariate Cox regression analysis for overall survival.

Variable	HR (95% CI)	*p* Value
Age ≥ 80 years	1.77 (1.15–2.74)	0.010
Albumin ≤ 40 g/L	1.11 (0.74–1.69)	0.609
ALP > 130 U/L	1.43 (0.97–2.11)	0.070
BPI/EQ-5D composite positive	1.54 (1.06–2.25)	0.025
Composite tumour-burden level 1	2.62 (1.20–5.69)	0.015
Composite tumour-burden level 2	5.52 (2.52–12.11)	<0.001
Hb < 120 g/L	1.52 (1.00–2.32)	0.052
LDH > 240 U/L	1.39 (0.88–2.20)	0.155
NLR > 2.5	1.50 (1.03–2.20)	0.036
PSA ≥ 100 ng/mL	1.34 (0.92–1.94)	0.128
Time since first prior oncological therapy <2 years	1.91 (1.33–2.75)	<0.001

HRs were estimated using a multivariate Cox proportional-hazards model. Reference categories were the complementary lower-risk categories for each binary variable; level 0 was the reference category for composite tumour-burden level. The model included 264 patients with complete covariate data and 131 deaths. Overall model fit: chi-square = 115.806, df = 11, *p* < 0.001. Harrell’s C-index was 0.779 (95% CI, 0.741–0.818). **Abbreviations:** ALP, alkaline phosphatase; BPI, Brief Pain Inventory; CI, confidence interval; EQ-5D, EuroQol 5-Dimension questionnaire; Hb, haemoglobin; HR, hazard ratio; LDH, lactate dehydrogenase; NLR, neutrophil-to-lymphocyte ratio; PSA, prostate-specific antigen.

**Table 4:** Prognostic components and risk-group classification of the MIRA score.

Score Component	Criterion	Points
Composite tumour-burden level	Level 0	0
	Level 1	1
	Level 2	2
Age	≥80 years	1
BPI/EQ-5D composite	Positive	1
Time since first prior oncological therapy	<2 years	2
NLR	>2.5	1
ALP	>130 U/L	1
Hb	<120 g/L	1
PSA	≥100 ng/mL	1

Composite tumour-burden level was assigned hierarchically. Level 2 was assigned to patients with ≥21 bone metastases or RECIST SLD >3 cm. Among the remaining patients, level 1 was assigned to those with 5–20 bone metastases or any RECIST target lesion. Level 0 was assigned to those with <5 bone metastases and no RECIST target lesion. Low risk was defined as 0–2 points, intermediate risk as 3–4 points and high risk as ≥5 points. **Abbreviations:** ALP, alkaline phosphatase; BPI, Brief Pain Inventory; CI, confidence interval; EQ-5D, EuroQol 5-Dimension questionnaire; Hb, haemoglobin; MIRA, Metastatic Integrated Risk Assessment; NLR, neutrophil-to-lymphocyte ratio; OS, overall survival; PSA, prostate-specific antigen; RECIST, Response Evaluation Criteria in Solid Tumours; SLD, sum of longest diameters.

**Table 5:** Comparison of MIRA and Halabi three-tier risk classifications.

Risk Classification	Risk Group and Cut-Off, Points	Events/Total (%)	Median OS (95% CI), Months	Harrell’s C-Index (95% CI)
**MIRA score**	Low risk, 0–2	6/69 (8.7)	Not reached	0.755 (0.722–0.788)
	Intermediate risk, 3–4	36/87 (41.4)	22.97 (22.21–23.23)	
	High risk, 5–9	90/109 (82.6)	11.56 (10.09–14.16)	
**Halabi score**	Low risk, <140	52/143 (36.4)	27.14 (22.57–not reached)	0.646 (0.603–0.690)
	Intermediate risk, 140–194.96	59/96 (61.5)	18.17 (14.92–22.54)	
	High risk, >194.96	22/27 (81.5)	9.72 (6.60–14.16)	

**Abbreviations:** CI, confidence interval; MIRA, Metastatic Integrated Risk Assessment for mCRPC; OS, overall survival. Survival distributions differed significantly across risk groups for both classifications by the log-rank test (*p* < 0.001). Harrell’s C-index values apply to the corresponding three-tier risk classification. Halabi risk groups were assigned using the published nomogram point cut-offs [1].

## Data Availability

The individual patient-level data analysed in this study were obtained from the publicly accessible Project Data Sphere repository and are available through the platform subject to registration and applicable data-access requirements. The dataset corresponds to D4320C00014/ENTHUSE-M1 (NCT00554229).

## Supplementary Materials

The following supporting information can be downloaded at: https://www.mdpi.com/article/10.3390/cancers18162660/s1. Figure S1: Bootstrap-corrected calibration of the final multivariate Cox model at (A) 12 months and (B) 24 months. Predicted survival probabilities are plotted against observed survival probabilities. The diagonal line represents perfect calibration. Calibration estimates were obtained using 1000 bootstrap resamples; Table S1: Frequency and handling of missing baseline data for candidate prognostic predictors with incomplete observations.
