# Supplementary material for: Incorporating Metastatic Disease Burden into Prognostic Assessment in Metastatic Castration-Resistant Prostate Cancer: The MIRA Score
Source: Cancers (Basel). 2026 Aug 18;18(16):2660. doi: 10.3390/cancers18162660 (PMC13511477; doi:10.3390/cancers18162660)
Supplement: Supplementary file 1 [file cancers-18-02660-s001.zip › cancers-4473710-resubmitted-supplementary.pdf]

Supplementary Table S1. Frequency and handling of missing baseline data for candidate prognostic predictors with incomplete observations.

Variable	Baseline available, n (%)	Post-baseline value used, n (%)	Timing of substitution, range (days)	Unavailable, n (%)
Albumin	265 (99.6)	1 (0.4)	29	0
ALP	265 (99.6)	1 (0.4)	29	0
First oncological therapy interval	265 (99.6)	N/A	N/A	1 (0.4)
Hb	264 (99.2)	2 (0.8)	29–57	0
LDH	259 (97.4)	7 (2.6)	27–61	0
Lymphocytes	264 (99.2)	2 (0.8)	29–57	0
Neutrophils	264 (99.2)	2 (0.8)	29–57	0
Platelets	261 (98.1)	4 (1.5)	29–57	1 (0.4)
PSA	262 (98.5)	4 (1.5)	6–91	0
RECIST status	265 (99.6)	N/A	N/A	1 (0.4)

All percentages were calculated using the full cohort of 266 patients. When a baseline laboratory value was unavailable, the earliest subsequent measurement was used. Six unique patients had a post-baseline value used for at least one laboratory component of the MIRA score and were excluded from the sensitivity analysis. The exact MIRA score was unavailable for two patients because of missing RECIST status or first oncological therapy interval. One of these patients could nevertheless be assigned unequivocally to the high-risk group, leaving one patient without a MIRA risk-group classification. First oncological therapy interval refers to the time since first prior oncological therapy.

Abbreviations: ALP, alkaline phosphatase; Hb, haemoglobin; LDH, lactate dehydrogenase; MIRA, Metastatic Integrated Risk Assessment; N/A, not applicable; PSA, prostate-specific antigen; RECIST, Response Evaluation Criteria in Solid Tumours.
